# Extralobar pulmonary sequestration: A case report and literature review

**DOI:** 10.1002/ccr3.8282

**Published:** 2023-12-05

**Authors:** Tao Wang, Zonglei Zhao, Lingqun Kong, Xiaoqin Lyu, Xuefeng Cao, Xingyuan Zhang, Qiangpu Chen

**Affiliations:** ^1^ Department of Hepatobiliary Surgery Binzhou Medical University Hospital Binzhou Shandong China

**Keywords:** case report, congenital lung anomaly, preoperative diagnosis, pulmonary sequestration, retroperitoneal mass

## Abstract

Pulmonary sequestration is a congenital malformation of lung development in which part of the lung tissue is separated from the normal lung during the embryonic phase and develops separately and receives blood supply from an aberrant systemic artery forming a nonrespiratory mass. In brief, early in embryonic development, certain tissues that should have atrophied and been gradually absorbed are left behind due to impairment of the atrophy process and form anomalous branches of the aorta, which pull parts of the lung tissue, isolating them from normal lung tissue and bronchi, and thus forming separate lung tissue. According to the relationship of the mass to the pleural covering, pulmonary sequestration can be divided into two types, intralobar pulmonary sequestration (ILS) and extralobar pulmonary sequestration (ELS), of which approximately 75% of cases are ILS, but ELS is less common. Symptoms are not obvious in either type, making diagnosis and differential diagnosis more difficult. Here we report a 33‐year‐old patient with only insignificant abdominal distension who was eventually diagnosed with retroperitoneal ELS.

## Graphical abstract

Pulmonary sequestration is a rare condition with no obvious symptoms, making it difficult to diagnose accurately. However, studying relevant cases can help to improve early diagnosis rates.

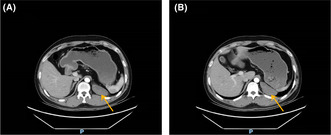

## 1 INTRODUCTION

**FIGURE 1:**
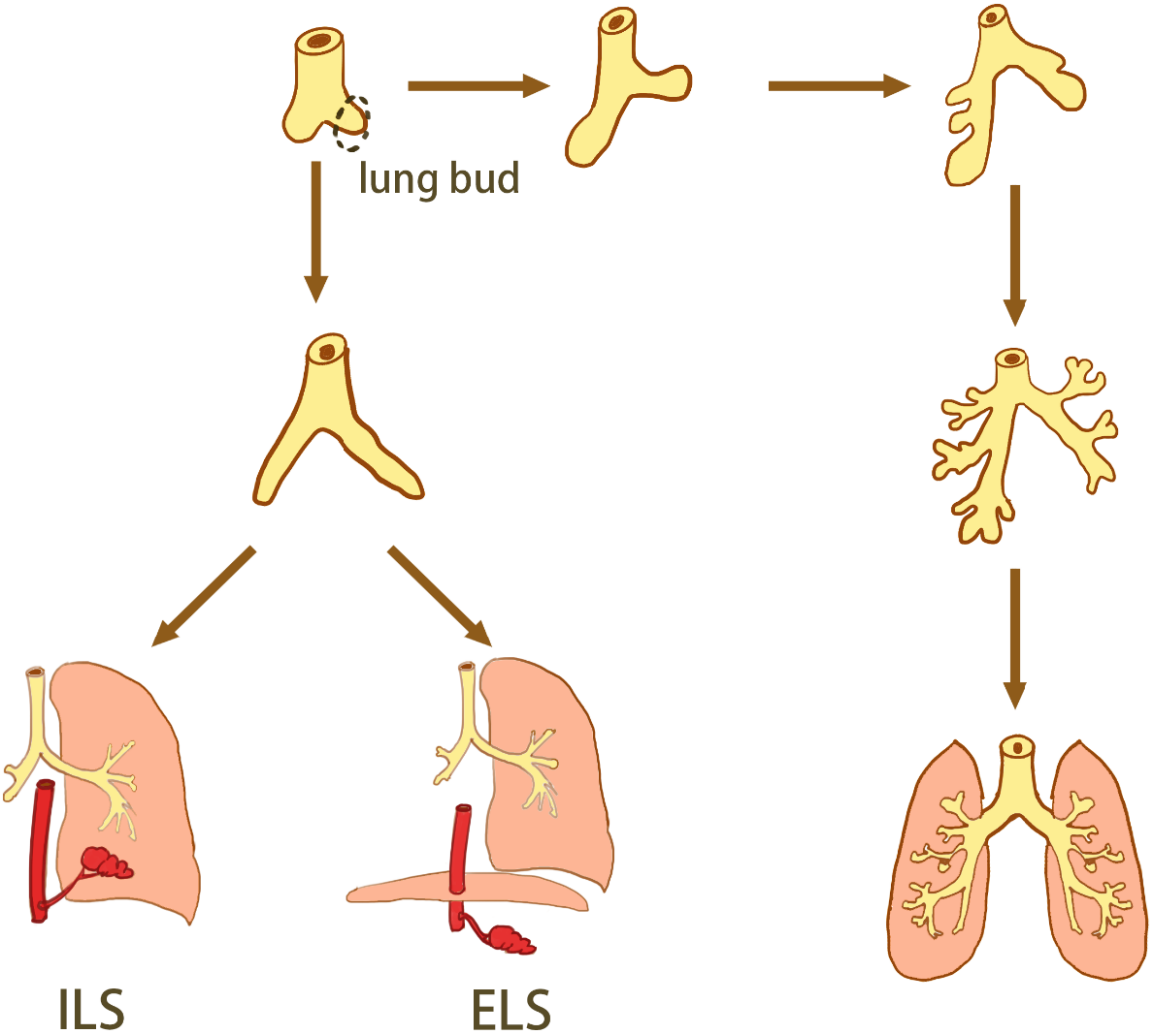
The development of pulmonary sequestration.

The pathogenesis of pulmonary sequestration (PS) is unclear and is currently considered to be common with the parapneumonic bud theory, the traction theory, and vascular development theory, but the parapneumonic bud theory is currently recognized, which anatomically classifies pulmonary sequestration into two types (Figure 1), intralobar pulmonary sequestration (ILS) and extralobar pulmonary sequestration (ELS), based on the relationship between the mass and the pleural covering.^1^ PS arises from the primitive foregut, if the pulmonary bud develops before the pleura and is covered by adjacent lung tissue, ILS is present and if this pulmonary bud develops later than the pleural development and grows independently, it results in ELS.^2^

The incidence of PS in congenital malformation of the lung is 1.1%–1.8%.^1^ ELS can occur in the thoracic cavity, mediastinum, pericardium, and intra‐ or sub‐diaphragm,^3^ and the retroperitoneal ELS of the 33‐year‐old man mentioned in this case is even rarer, with only 10%–15% of ELS being found in retroperitoneal sites.^4^

## 2 CASE REPORT

**FIGURE 2:**
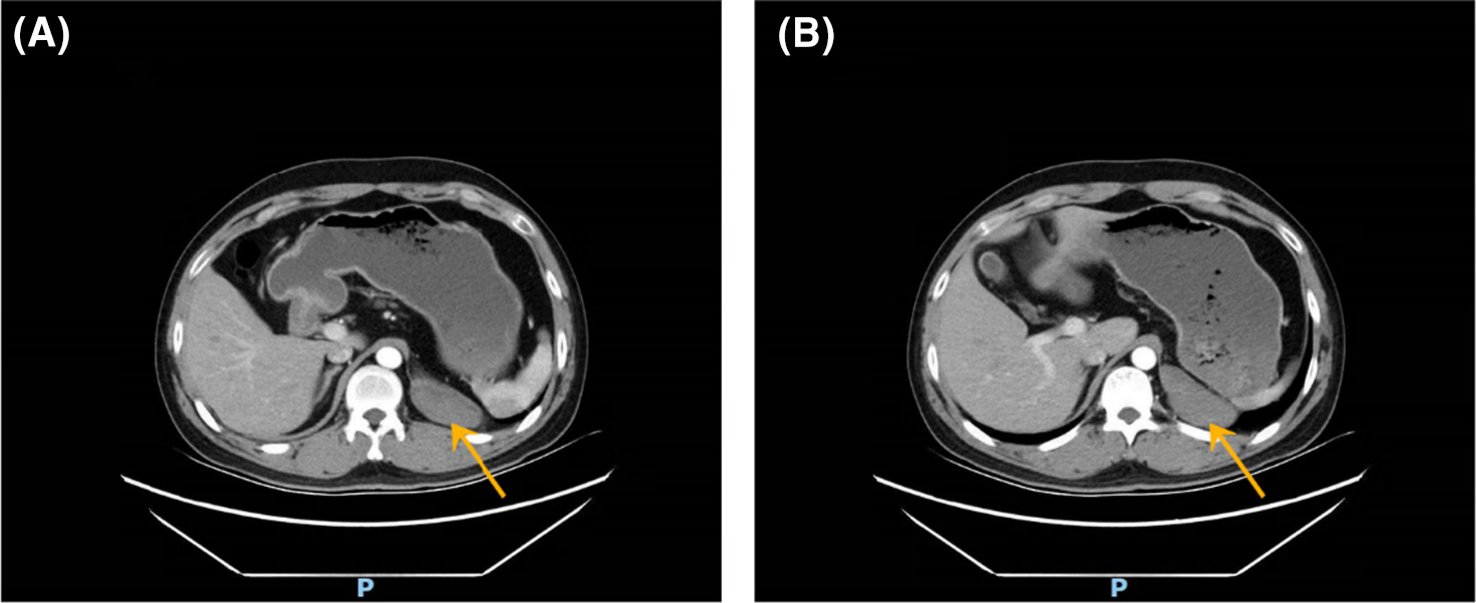
Computed tomography scan of the abdomen showing a well‐delineated soft tissue structure measuring 7.5 × 2.5 cm in the left diaphragm area, (A and B) are both arterial phase images, showing the mass at different levels, which may indicate the suspected blood supply artery from the abdominal aorta. An orange arrow has been used to mark the location of the mass.

**FIGURE 3:**
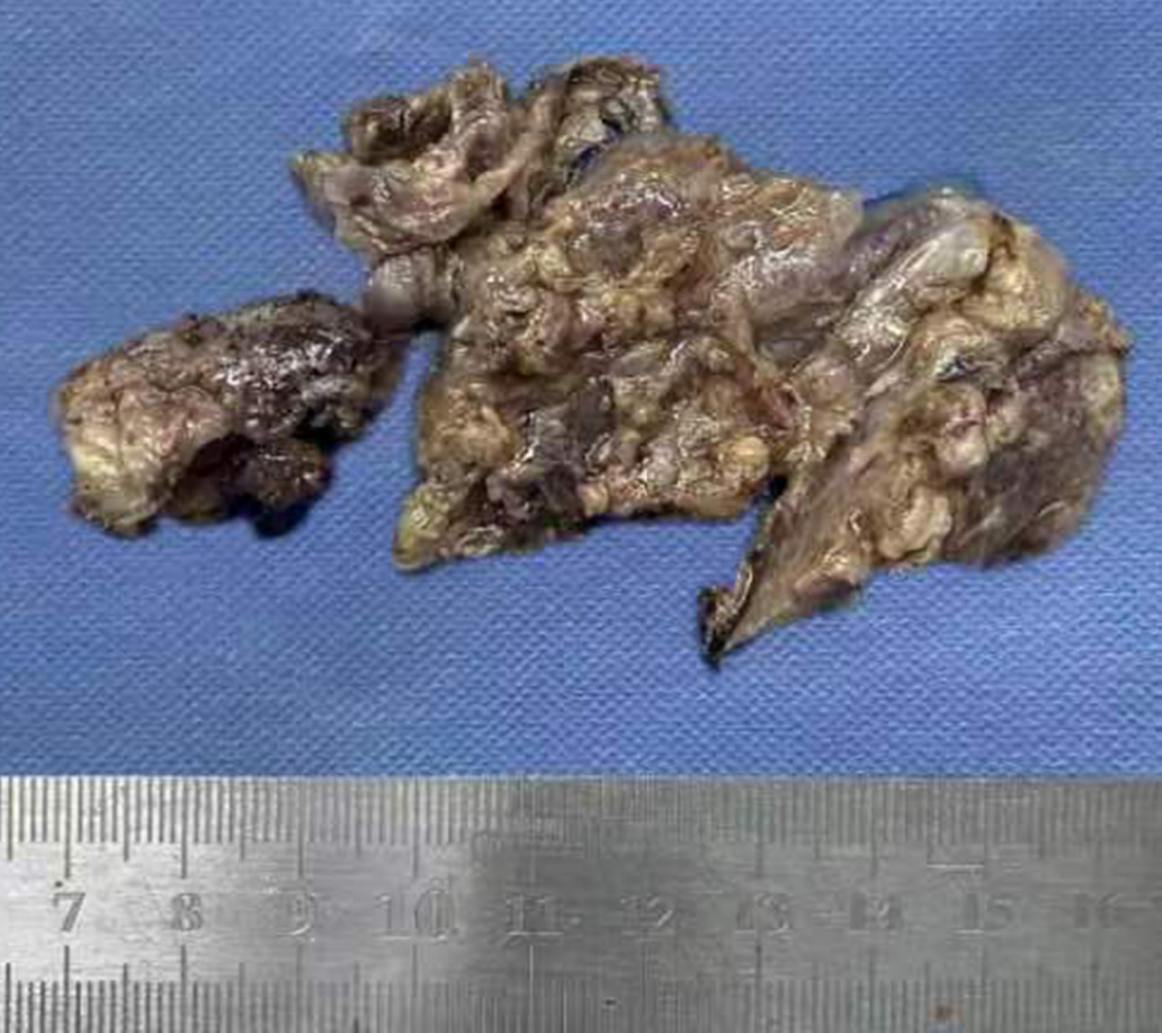
Macroscopy revealed a multicystic mass with gray–red color, measuring 6.5 × 4 × 3 cm.

**FIGURE 4:**
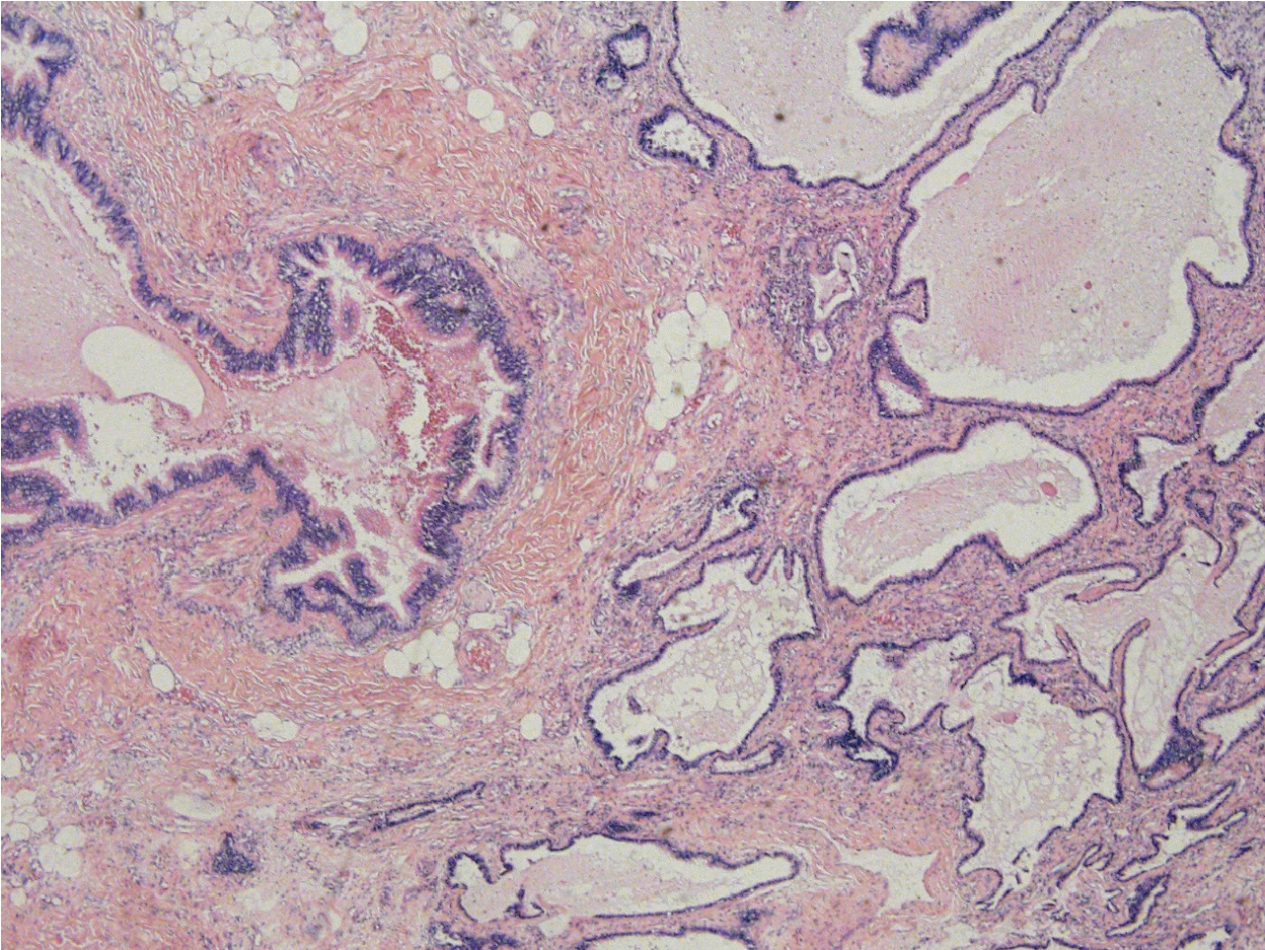
Pathology image.

A 33‐year‐old man was admitted to our hospital for a retroperitoneal mass. He had been admitted to a local hospital for abdominal distension and computed tomography (CT) of the abdomen revealed an space‐occupying lesion in the left diaphragm. The patient only felt abdominal distension and had no other significant abnormalities in his physical examination, medical history, or family history. The patient reported that he was prone to upper respiratory tract infections when he was young and had been misdiagnosed with “mycoplasma pneumonia.” He also felt chest tightness and dyspnea after exertion, and the left side of his face and left ear were easily flushed, often with acid reflux and heartburn. However, these symptoms disappeared in adulthood. Previously, a malignant tumor of the abdominal cavity, retroperitoneal schwannoma was suspected. The patient's vital signs were stable and laboratory tests showed aldosterone 60.21 pg/mL, and the rest of the indicators were within normal limits. CT of the upper abdomen and chest revealed a well‐delineated, soft tissue, dense structure measuring 7.5 × 2.5 cm that was located in the left diaphragm and the suspected blood supply artery from the abdominal aorta could be identified on CT (Figure 2). Ultrasound‐guided puncture biopsy showed little fibrous connective tissue and mucus, and the diagnosis was not clear before surgery. Laparoscopic exploration was performed, and the intraoperative mass was seen to be a multicystic mass, measuring 10 × 5 × 4 cm, containing black mucus, reaching posteriorly to the diaphragm and inferiorly to the superior margin of the pancreas, with an extensive base and a deep location. The upper edge of the mass was seen to be tightly adherent to the pleura, and its base was deeper and more heavily adherent to the diaphragm. Therefore, laparotomy was performed, and the mass was peeled away from the surrounding tissues and excised completely. Macroscopy revealed a multicystic mass with a gray–red color, and when the resection was completed, the mucus contained in the mass was drained (Figure 3). The pathological diagnosis was retroperitoneal ELS (Figure 4). The patient recovered uneventfully and was discharged on postoperative Day 8.

## 3 DISCUSSION

PS is very rare, with a prevalence of 0.225%–0.425%, and ELS is less common than ILS, with most ELS located between the left lower lobe and the septum.^5^ However, the mass in our patient was located on the left side of the diaphragm and was usually asymptomatic or mildly symptomatic.

Due to the presence of the pleura, lobar lung isolation is completely separated from the adjacent normal lung tissue anatomically and forms a separate lung lobe. Ninety percent of ELS is located in the left hemithorax,^1^ and rarely exists in extrathoracic locations.^6^ PS receives blood supply from the systemic circulation, most commonly from the thoracic or abdominal aorta, with venous drainage to the pulmonary veins, the azygous vein, the hemiazygos vein, the inferior vena cava, or the right atrium.^7^ In contrast, the suspected supply artery from the abdominal aorta could be identified in the imaging presentation of this patient. Abdominal PS can present as a retroperitoneal mass or as a cyst, which can be located next to or in communication with the stomach.^8^ Retroperitoneal pulmonary sequestration (RPS) is a rare cause of retroperitoneal cysts or masses that cannot be well identified.^9^ As the diagnosis of RPS in this patient was difficult to make preoperatively, the diagnosis could only be confirmed by intraoperative excision of tissue for pathological examination.

The diagnosis of PS can be accomplished with CT angiography. CT angiography provides a noninvasive method compared to catheter aortography, which used to be necessary to determine the arterial supply and make the diagnosis.^7^ However, in our cases, it was very difficult to make a correct preoperative diagnosis from the imaging presentation. A large part of the reason for this is the rarity of the disease, and our first consideration in clinical work is definitely the most common disease.

Through our systematic review of the relevant literature in recent years (Table 1), we have listed 17 cases in which the masses occurred in different locations, mostly on the left side, and with different clinical presentations. The cases we have listed cover not only patients of all ages but also several common types of disease. Based on these cases, we can generally conclude that the majority of RPS occurs close to the left adrenal region and that the nature of the mass is difficult to confirm by imaging alone, whereas PS occurring at other sites can be correctly diagnosed preoperatively by imaging and that the location and morphologic characteristics of the mass are more reliably defined during surgical exploration for RPS. The diagnosis can be clarified by pathological testing after removal of the mass. On the other hand, regardless of the type of PS, the treatment is currently based on surgical interventions, but if the patient has no obvious symptoms and if the PS has no impact on the patient's life, then leaving the mass untreated could be considered. However, in most cases, surgical resection is needed. Intraoperative care should be taken to avoid damage to nerves and blood vessels and to reduce postoperative complications, and in most cases, the outcome of surgical resection is curative. Reviewing the relevant literature, with the intensive research on PS in recent years, the treatment modalities have become diversified and endovascular treatment is another option available. This less invasive treatment modality has fewer complications.^22^ Currently a thoracic endograft can be used for PS.^23^ We can consider thoracic endograft as a first‐line treatment because this treatment has the least physiological burden on the patient and the fastest recovery^24^ or we can perform vascular embolization with Glubran (n‐butyl cyanoacrylate), an endovascular technique that may be a less invasive alternative for patients with comorbidities or who have refused surgery.^25^

**TABLE 1:** Summary of PS cases in the current literature.

Authors	Published year	Age	Sex	Main symptom	Location	Side	Complication	Resection
Liu^1^	2012	74	M	Asymptomatic	Adrenal area	Left	–	Yes
Sagir Khan^3^	2019	67	F	Asymptomatic	Adrenal area	Left	–	Yes
Sagir Khan^5^	2020	10	M	Chest pain and vomiting	Thoracic cavity	Left	Torsion	Yes
Petty^7^	2017	43	M	Back pain	Lower lobe	Left	–	Yes
Armatys^9^	2005	21	F	Flank pain	Adrenal area	Left	Pneumothorax and a diaphragmatic injury	Yes
Marine^10^	2022	51	F	Chest pain and hemoptysis	Lower lobe	Right	–	No
Gupta^11^	2017	28	M	Postprandial epigastric pain	Lower lobe	Left	–	Yes
Hakiri^12^	2021	56	F	chest pain	Lower lobe	Left	–	Yes
Son^13^	2020	13	F	Abdominal pain and fever	Thoracic cavity	Left	Torsion	Yes
Jaiswal^14^	2021	22	M	Massive haemoptysis	Lower lobe	Left	–	Yes
Baker^15^	1982	50	M	Urinary tract infection	Adrenal area	Left	–	Yes
Gomez^16^	2009	20 day	M	–	Below the diaphragm, in front of the aorta, and between the right kidney and stomach	Right	‐	Yes
Gucer^17^	2006	5	M	Abdominal pain and weight loss	Adrenal area	Left	–	Yes
Roberts^18^	2000	74	F	Acute gastroenteritis	Adrenal area	Left	–	Yes
Furuno^19^	2006	41	M	Flank pain	Adrenal area	Left	–	Yes
Kim^20^	2005	32	M	Epigastric discomfort and	Retroperitoneal space	Left	Infection	Yes
Yang^21^	2012	40	M	Flank pain	Adrenal area	Left	–	Yes

## 4 CONCLUSION

PS can have many different symptoms and can occur in different locations, requiring attention to the differential diagnosis. Similar to the RPS mentioned in this case, we can only make a preliminary diagnosis of a retroperitoneal mass. Reviewing the relevant literature, we learned that bronchogenic cysts,^26^ retroperitoneal lipomas,^27^ retroperitoneal dendritic cell sarcoma,^28^ and retroperitoneal ganglioneuromas^29^ all have imaging manifestations of retroperitoneal masses and require careful differentiation. The uniqueness of the case is that the symptoms of RPS are only inconspicuous rather than asymptomatic, and because we had so little knowledge of pulmonary isolation, we were unable to make an accurate preoperative diagnosis. The outcome of surgical resection was curative, as determined through long‐term postoperative follow‐up of the patient.

## AUTHOR CONTRIBUTIONS

**Tao Wang:** Writing – original draft. **Zonglei Zhao:** Resources; writing – review and editing. **Lingqun Kong:** Resources; writing – review and editing. **Xiaoqin Lyu:** Writing – review and editing. **Xuefeng Cao:** Writing – review and editing. **Xingyuan Zhang:** Writing – review and editing. **Qiangpu Chen:** Writing – review and editing.

## FUNDING INFORMATION

This research was supported by the Project of Medical and Health Technology Development Program in Shandong Province (202104081018).

## CONFLICT OF INTEREST STATEMENT

The authors declare that they have no conflict of interest.

## ETHICS STATEMENT

The study was approved by the Ethics Committee of Binzhou Medical University Hospital. Informed consent can be obtained from the clinical database of hospital.

## CONSENT

Written informed consent was obtained from the patient to publish this report in accordance with the journal's patient consent policy.

## Data Availability

The data that support the findings of this study are available from the corresponding author upon reasonable request.

## References

[1] Liu L , Han P , Zhu Y , et al. Intra‐abdominal pulmonary sequestration: a case report and literature review. Urol Int. 2012;88(1):121‐124. doi:10.1159/000331688 21967921

[2] Gabelloni M , Faggioni L , Accogli S , Aringhieri G , Neri E . Pulmonary sequestration: what the radiologist should know. Clin Imaging. 2021;73:61‐72. doi:10.1016/j.clinimag.2020.11.040 33310586

[3] Sagir Khan I , Chua D , Wong B , Wang S , Nga ME . Intra‐abdominal pulmonary sequestration: a rare diagnostic pitfall on EUS‐FNA. Pathology. 2019;51(7):747‐750. doi:10.1016/j.pathol.2019.08.008 31668404

[4] Zhao O , Zhang C , Lv F , Wu Y . Prenatal detected retroperitoneal pulmonary sequestration with elevated serum levels of CA 19‐9—case report and review of the literature. J Pediatr Surg Case Rep. 2013;1(4):68‐70.

[5] Yang L , Yang G . Extralobar pulmonary sequestration with a complication of torsion: a case report and literature review. Medicine (Baltimore). 2020;99(29):e21104. doi:10.1097/MD.0000000000021104 32702859 PMC7373605

[6] Ryujin K , Akamine T , Miura N , et al. An adult case of multiple extralobar pulmonary sequestrations in the thoracic and abdominal cavities. Ann Thorac Surg. 2022;113(1):e17‐e20. doi:10.1016/j.athoracsur.2021.03.083 33839125

[7] Petty L , Joseph A , Sanchez J . Case report: Pulmonary sequestration in an adult. Radiol Case Rep. 2017;13(1):21‐23. doi:10.1016/j.radcr.2017.09.029 29487633 PMC5826459

[8] Case records of the Massachusetts General Hospital. Weekly clinicopathological exercises. Case 36‐1999. A 32‐day‐old girl with a retroperitoneal mass. N Engl J Med. 1999;341(22):1680‐1685. doi:10.1056/NEJM199911253412208 10572157

[9] Armatys SA , Cheng L , Gardner TA , Sundaram CP . Pulmonary sequestration presenting as retroperitoneal cyst: case report. J Endourol. 2005;19(8):997‐999. doi:10.1089/end.2005.19.997 16253068

[10] Marine L , Torrealba JI , Valdes F , et al. Endovascular treatment of a right pulmonary sequestration supplied by an aneurysmal aberrant artery originating from the abdominal aorta. J Vasc Bras. 2022;23(21):e20190160. doi:10.1590/1677-5449.201901602. PMC9136689 35677746

[11] Gupta R , Patadia D , Belligund P . An atypical presentation of pulmonary sequestration. J Res Med Sci. 2017;22:127. doi:10.4103/jrms.JRMS_234_17 29259638 PMC5721488

[12] Hakiri S , Fukui T , Chen‐Yoshikawa TF . Combined surgical therapy for pulmonary sequestration and aberrant artery from the abdominal aorta. Gen Thorac Cardiovasc Surg. 2021;69(6):1031‐1034. doi:10.1007/s11748-021-01612-6 33743137

[13] Son SA , Do YW , Kim YE , Lee SM , Lee DH . Infarction of torsed extralobar pulmonary sequestration in adolescence. Gen Thorac Cardiovasc Surg. 2020;68(1):77‐80. doi:10.1007/s11748-019-01105-7 30875002

[14] Jaiswal LS , Neupane D . Pulmonary sequestration presenting as a massive haemoptysis in adult: a case report. Int J Surg Case Rep. 2021;86:106341. doi:10.1016/j.ijscr.2021.106341 34488138 PMC8424506

[15] Baker EL , Gore RM , Moss AA . Retroperitoneal pulmonary sequestration: computed tomographic findings. AJR Am J Roentgenol. 1982;138(5):956‐957. doi:10.2214/ajr.138.5.956 6979187

[16] Gomez L , Robert JA , Sepulveda W . Fetal retroperitoneal pulmonary sequestration with an atypical vascular pattern. Prenat Diagn. 2009;29(3):290‐291. doi:10.1002/pd.2200 19194869

[17] Gucer S , Caliskan U , Ucar C , Koksal Y , Dilsiz A , Kale G . A suprarenal mass in a child. Eur J Pediatr. 2006;165(10):736‐738. doi:10.1007/s00431-006-0149-5 16912900

[18] Roberts WW , Nelson JB , Fishman EK , Jarrett TW . Diagnosis of retroperitoneal pulmonary sequestration using computerized tomography guided fine needle biopsy. J Urol. 2000;164(2):445. 10893609

[19] Furuno T , Morita K , Kakizaki H , Harabayashi T , Watarai Y , Nonomura K . Laparoscopic removal of a retroperitoneal extralobar pulmonary sequestration in an adult. Int J Urol. 2006;13(2):165‐167. doi:10.1111/j.1442-2042.2006.01251.x 16563142

[20] Kim HK , Choi YH , Ryu SM , et al. Infected infradiaphragmatic retroperitoneal extralobar pulmonary sequestration: a case report. J Korean Med Sci. 2005;20(6):1070‐1072. doi:10.3346/jkms.2005.20.6.1070 16361825 PMC2779312

[21] Yang HJ , Lee SW , Lee HJ , Lee JH , Jeon YS . Extralobar pulmonary sequestration mimicking an adrenal tumor. JSLS. 2012;16(4):671‐674. doi:10.4293/108680812X13517013316834 23484585 PMC3558913

[22] Belczak SQ , da Silva IT , Bernardes JC , et al. Pulmonary sequestration and endovascular treatment: a case report. J Vasc Bras. 2019;10(18):e20180110. doi:10.1590/1677-5449.011018 PMC6542322 31191630

[23] Chen Y , Liu B , Shao J , Liu D , Zheng Y . Endovascular treatment of pulmonary sequestration with thoracic endograft: two case reports. Medicine (Baltimore). 2019;98(31):e16666. doi:10.1097/MD.0000000000016666 31374041 PMC6708911

[24] Wilder FG , Minasyan SZ . Thoracic stent graft accompanied by coil embolization for pulmonary sequestration. Innovations (Phila). 2019;14(2):168‐173. doi:10.1177/1556984519827695 31039681

[25] Szmygin M , Pyra K , Sojka M , Jargiełło T . Successful endovascular treatment of intralobar pulmonary sequestration‐an effective alternative to surgery. Pol J Radiol. 2021;15(86):e112‐e114. doi:10.5114/pjr.2021.103975 PMC7976228 33758636

[26] Hu BY , Yu H , Shen J . A retroperitoneal bronchogenic cyst clinically mimicking an adrenal mass: three case reports and a literature review. J Int Med Res. 2022;50(1):3000605211072664. doi:10.1177/03000605211072664 35023387 PMC8785309

[27] Petca RC , Ambert V , Popescu RI , et al. Half abdomen tumor‐giant retroperitoneal lipoma: a case report and review of the literature. Rom J Morphol Embryol. 2022;63(1):237‐244. doi:10.47162/RJME.63.1.27 36074690 PMC9593116

[28] Wang C , Lei P , Wan Y , et al. Retroperitoneal dendritic cell sarcoma: a case report. Medicine (Baltimore). 2021;100(9):e24459. doi:10.1097/MD.0000000000024459 33655917 PMC7939173

[29] Kirchweger P , Wundsam HV , Fischer I , et al. Total resection of a giant retroperitoneal and mediastinal ganglioneuroma—case report and systematic review of the literature. World J Surg Oncol. 2020;18(1):248. doi:10.1186/s12957-020-02016-1 32948207 PMC7501651

